# Designing an Accurate Temperature Control System for Infrared Earth Simulators Using Semiconductor and Air Cooling Integration

**DOI:** 10.3390/s23156908

**Published:** 2023-08-03

**Authors:** Jiachong Li, Lingyun Wang, Guangxi Li, Sida Mu

**Affiliations:** School of Optoelectronic Engineering, Changchun University of Science and Technology, Changchun 130022, China; lijiachong126@163.com (J.L.); liguangxi@mails.cust.edu.cn (G.L.); mstardcl@163.com (S.M.)

**Keywords:** Earth simulator, temperature analysis, fuzzy controls, temperature control

## Abstract

In a laboratory environment, in order to test the attitude recognition capability and accuracy of the satellite attitude sensor—the infrared Earth sensor—the infrared Earth simulator is fixed on a five-axis turntable to enable multi-angle testing. In the past, the temperature control system of the Earth simulator was water cooled, which not only affected the working accuracy of the Earth simulator but also affected its size and portability and made it more difficult to use on the turntable. Therefore, we designed a cooling method for the cold plate based on semiconductor cooling technology combined with air cooling, and we designed a fuzzy PID control algorithm to accurately control the temperature according to this cooling method. In this article, we use SOLIWORKS to build the system model for the system and use the ANAYS Workbench to perform temperature analysis of the Earth simulator. The results show that the cold plate temperature can be maintained at 20.089 °C when the hot plate temperature is 85 °C. The overall temperature uniformity of the hot plate is better than ±0.3 °C, which meets the index requirements of the Earth simulator. We found that this cooling method can replace water cooling, giving the simulator the advantage of being miniaturized, and it can be adaptable to the turntable, which can be widely used in various sizes of Earth simulators and in various complex environments and operating conditions.

## 1. Introduction

The infrared Earth sensor is an important attitude measurement component for satellites in space [1] that takes the Earth as the target source for the satellite attitude’s reference. Different attitude information of the satellite with respect to the Earth is obtained by means of infrared optical detection, which enables the measurement of satellite roll and pitch attitude deviation angles [2].

The infrared Earth simulator is a special piece of ground performance test equipment for infrared Earth sensors, which is a necessary means of realizing the ground test evaluation of the sensor’s performance index; the simulation accuracy of the Earth simulator directly affects the performance test of the sensors. The United States first successfully developed an infrared Earth simulator, followed by Italy, which developed two Earth simulators that can simulate the height of geosynchronous orbit. France and other European and American countries have also completed the development of Earth simulators. The China Aerospace Science and Technology Corporation developed an Earth simulator with an aperture of 250 mm. Changchun University of Science and Technology [3] developed an Earth simulator that can change according to the orbital altitude, and the Shanghai Institute of Technology Physics [4] has also made breakthroughs in the development of Earth simulators. However, the infrared Earth simulators studied above still have more problems, and all of their cooling links are constructed with water cooling. Because the structure of water-cooled radiators is very large, it takes up a lot of space. And, as the water-cooled structure is complex and costly, there is a risk of damaging system parts if water leaks. In addition, water cooling also leads to the inability to control the temperature precisely, which in turn affects the overall working of the Earth simulator.

To improve the testing accuracy of Earth sensors, we propose to use the simulator on a turntable. And, for the problems of excessive volume, poor working reliability, and low temperature control accuracy of Earth simulators, semiconductor cooling combined with air cooling is proposed to replace the traditional water cooling system and to optimize the temperature control system of Earth simulators. The cold plate temperature of the simulators is maintained at 20 °C, the temperature of the Earth hot plate is continuously adjustable between 35 °C and 85 °C, and the temperature uniformity of the hot plate is guaranteed to be better than ±0.5 °C. This opens up the development of Earth simulators and provides a new idea for it.

The rest of this paper is organized as follows. We will first introduce the infrared Earth simulator composition and the working principle. In Section 3, we design the Earth simulator temperature control system. Firstly, the temperature control range of the Earth hot plate is calculated, and secondly, a fuzzy PID control algorithm is designed and verified; finally, the specific parts of the Earth simulator are designed. Then, we validate the overall Earth simulator with experimental simulations in Section 4. Finally, we conclude in Section 5.

## 2. Infrared Earth Simulator Composition and Working Principle

**Figure 1:**
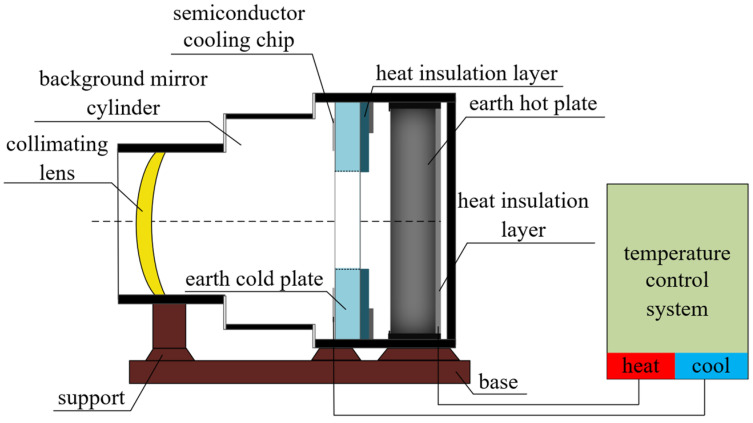
Infrared Earth simulator composition structure.

The infrared Earth simulator designed in this paper mainly consists of a collimating lens and its components, a background mirror cylinder, an Earth cold plate and its components, an Earth hot plate and its components, a temperature control system, a heat insulation layer, a base and support, etc. The overall structure of the Earth simulator is shown in Figure 1.

**Figure 2:**
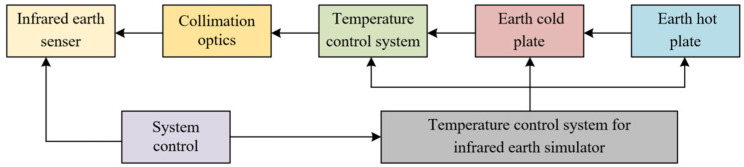
Infrared Earth simulator working principle.

The purpose of the infrared Earth simulator is to complete the simulation of the Earth on the ground so as to check the performance of the infrared Earth sensor and determine whether its technical parameters are up to standard. The infrared Earth sensor is a satellite attitude detection component that uses infrared thermal imaging principles to observe the Earth in space to determine the satellite’s attitude [5,6]. The Earth simulator needs to simulate the relevant states, such as the size of the horizon circle and the radiance difference of the Earth in space, and set the turntable parameters to solve for the difference between the satellite attitude parameters output by the Earth sensor and the attitude parameters set by the turntable. Then, it needs to set the parameters of the sensor so that the measurement error can be minimized. The working principle of the infrared Earth simulator designed in this paper is shown in Figure 2.

The core of the infrared Earth simulator design is the temperature control system, which aims to simulate the intensity of the Earth’s radiation in space in the laboratory. The temperature control system makes the radiance of the blackbody light source (i.e., the Earth hot plate) reach the radiance value of the Earth proper through the heating device, and then it makes the radiance of the Earth cold plate equal to the cosmic radiance value through the cooling device [7]. By controlling the relationship between the position of the hot plate and cold plate in the Earth simulator, the Earth as seen by a satellite in the background of space can be simulated in the laboratory.

## 3. Earth Simulator Temperature Control System Design

### 3.1. Temperature Determination

The Earth’s temperature varies with the season, the longitude, and the sunlight direction, and the Earth’s radiation varies [8,9]. In the 14 μm~16 μm band, the Earth’s radiance reaches the maximum (Arctic summer) N_max_ = 6.2 W/m^2^·Sr and the minimum (Arctic winter) N_min_ = 3.7 W/m^2^·Sr, so the Earth simulator should meet the radiation energy in this range. The radiation energy of the part outside the Earth cold plate simulates the radiative properties of the space background, and the radiative energy of the part inside the cold plate simulates the radiative properties of the Earth. The difference between the radiation energy of these two parts should be the radiated energy obtained by the infrared Earth sensor when the satellite is operating in space, i.e., Earth’s radiation energy in real space [10].

**Figure 3:**
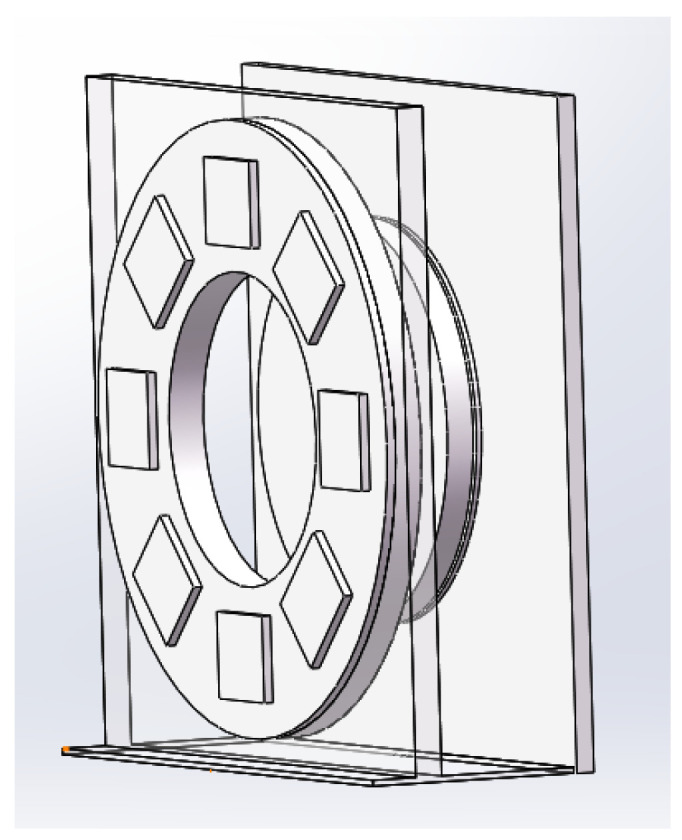
Structure of the Earth simulator.

As shown in Figure 3, the Earth simulator is placed in a laboratory with an ambient temperature of 20 °C. The temperature of the Earth cold plate is controlled at 20 °C. It is necessary to determine the infrared radiation energy of the Earth hot plate, i.e., to determine the temperature of the hot plate.

The radiation energy of a blackbody is calculated according to Planck’s radiation equation for its radiant exitance [11], as shown in Equation (1):

(1) $$M=\int_{\lambda_{1}}^{\lambda_{2}}\frac{C_{1}}{\lambda^{5}(e^{C_{2}/\lambda T}-1)}d\lambda$$

where λ λ denotes the wavelength, λ_1_$\lambda_{1}$ and λ_2_$\lambda_{2}$ denote the lower and upper limits of the wavelength, respectively, C_1_$C_{1}$ = 3.7418 × 10^−16^ W·m^2^ denotes the first radiation constant, C_2_ $C_{2}$ = 1.4338 × 10^−2^ m·K denotes the second radiation constant, and T denotes the temperature (K).

The radiation brightness can be calculated by the radiant exitance when, for the surface radiation, its radiation brightness is:

(2) $$N=\frac{M}{\pi }$$

**Figure 4:**
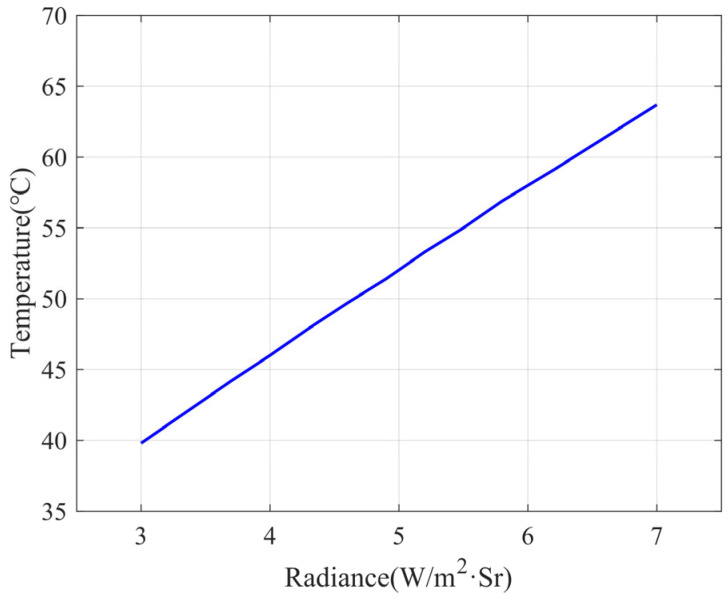
Radiance versus temperature.

The correspondence between the temperature of the hot plate and the actual Earth’s radiance calculated by MATLAB is shown in Figure 4. The radiant exitance is 38.9 W/m^2^, which corresponds to a radiance of 12.4 W/m^2^·Sr. When the radiance of the cold plate is taken to be 6.2 W/m^2^·Sr, the radiance of the hot plate is 18.6 W/m^2^·Sr, which corresponds to a radiant exitance of 58.4 W/m^2^, and the temperature of the hot plate is 332.25 K, or 59.1 °C. When the radiance of the cold plate is taken to be 3.7 W/m^2^·Sr, the corresponding hot plate temperature is 316.35 K, i.e., 44.2 °C. Considering the blackbody emissivity, and to ensure that the infrared Earth sensor receives enough Earth radiation energy, the temperature of the hot plate is designed to be continuously adjustable, the adjustment range is 35 °C~85 °C, and the temperature distribution is required to be uniform.

### 3.2. Temperature Control Algorithm

#### 3.2.1. Fuzzy PID Control Algorithm

Through the analysis of temperature changes in the Earth simulator after heating, it is known that the system is a large inertia and large hysteresis system; the dynamic characteristics are not easy to grasp, and it is difficult to establish an accurate mathematical model. Therefore, using only the traditional PID controller control system often leads to disadvantages, such as large system overshoot and a long transition time [12].

**Figure 5:**
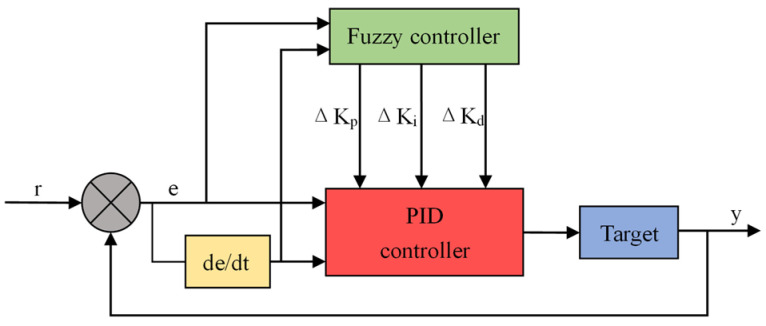
Fuzzy PID control principle diagram.

To compensate for these deficiencies, we use a fuzzy PID control algorithm in this paper. The essence is to obtain the best control performance of the system by automatically adjusting the PID parameters [13]. The deviation amount e and the deviation change ec are used as input variables, and $∆K_{p}$, $∆K_{i}$ and $∆K_{d}$ are output variables. By continuously detecting e and ec, the values of $∆K_{p}$, $∆K_{i}$, and $∆K_{d}$ are adjusted according to the fuzzy rules and then output to the PID controller to obtain three new parameters. The output quantity of the controller is continuously updated to finally find the best combination of $K_{p}$, $K_{i}$, and $K_{d}$ [14,15]. The principle of fuzzy PID control is shown in Figure 5.

According to the input r and the output y of the temperature control system, select e, ec with $K_{p}$, $K_{i}$, and $K_{d}$, whose fuzzy language variables are all [−6, 6] and whose fuzzy subsets are all {NB, NM, NS, ZO, PS, PM, PB}, and establish a fuzzy control rule table based on the control experience. For example, if the input quantity e is PB and the deviation change ec is NB at the same moment, this means that the difference between the actual temperature and the set target is great, but the error is rapidly decreasing and is not adjusted $K_{p}$. If e is PB and ec is NS, it means that the difference between the actual temperature and the set target is very large, and the change of error reduction is not obvious. Then, the heating power of the heating film needs to be reduced in order to reduce the process of increasing temperature error, so $K_{p}$ needs to be reduced appropriately.

In other cases, the fuzzy rules for $∆K_{p}$, $∆K_{i}$, and $∆K_{d}$ are determined along the same lines. According to the fuzzy rule table, dynamic rectification is performed for $K_{p}$, $K_{i}$, and $K_{d}$. Let ${K^{\prime }}_{p}$, ${K^{\prime }}_{i}$, and ${K^{\prime }}_{d}$ be the pre-tuned values of $K_{p}$, $K_{i}$, and $K_{d}$ obtained by using conventional methods [16]. Select appropriate fuzzification and defuzzification methods, and then the fuzzy PID parameters are:

(3) $$\left\{\begin{array}{l} K_{p}={K^{\prime }}_{p}+∆{K^{\prime }}_{p}\\ K_{i}={K^{\prime }}_{i}+∆{K^{\prime }}_{i}\\ K_{d}={K^{\prime }}_{d}+∆{K^{\prime }}_{d} \end{array}\right.$$

**Figure 6:**
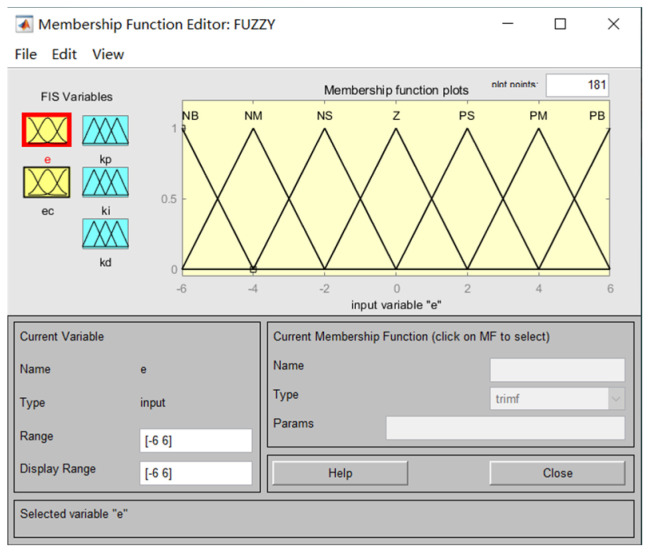
Affiliation function curve.

The affiliation function curves are shown in Figure 6.

According to the characteristics of the Earth simulator temperature control system, the first-order inertial pure hysteresis model is chosen to identify the system [17], and its mathematical model is:

(4) $$G(s)=\frac{Ke^{-\tau s}}{Ts+1}$$

The design uses an incremental PID control algorithm, whose output quantity u(t) u(t) is only related to the current cycle and the value of the first two cycles of the deviation quantity e(t) e(t), which is a simple algorithm that can achieve good control effects [18].

The digital PID incremental type control formula is:

(5) ∆u(k)=Kp[e(k)−e(k−1)]+Kie(k)+Kd[e(k)−2e(k−1)+e(e−2)]

That is:

(6) ∆u(k)=Kp∆e(k)+Kie(k)+Kd[∆e(k)−∆e(k−1)]

#### 3.2.2. MATLAB Simulation

**Figure 7:**
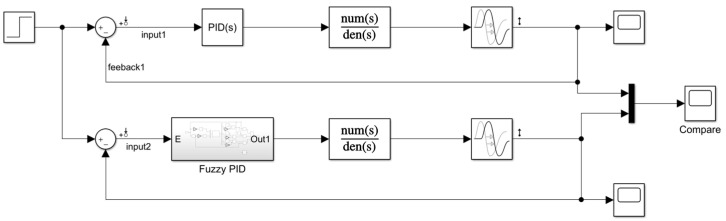
System model building.

To test whether the algorithm can effectively control the temperature variation of the Earth simulator, simulation tests are conducted using the Simulink part of MATLAB. According to the temperature control system, to establish a mathematical model, input the step signal, ignore other signal interference, compare the traditional PID controller and the fuzzy PID controller, respectively, input the signal processing, and observe the signal change at the output; the overall structure is shown in Figure 7.

**Figure 8:**
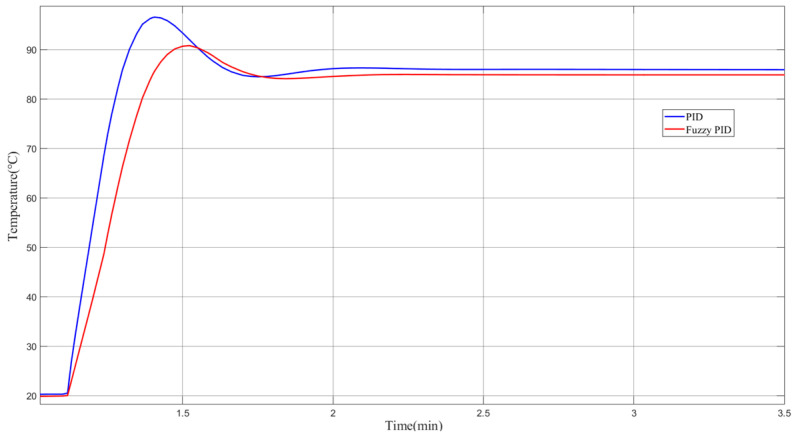
Fuzzy PID and traditional PID control response curve.

When the initial Earth hot plate temperature value is set to 20 °C and the target temperature is 85 °C (the temperature output curve of the Scope module in Simulink is shown in Figure 8), the blue line is the traditional PID control curve, and the red line is the fuzzy PID control effect curve. According to the Earth simulator temperature control system indicators, the traditional PID has a large amount of overshoot, a maximum temperature of more than 95 °C, and a fuzzy control of the maximum temperature of about 90 °C, and the amount of overshoot is reduced by 5 °C. The traditional PID temperature control is around 86 °C, and the fuzzy PID control is around 85 °C, which improves the accuracy by about 1 °C in comparison. And, the response time is about 0.5 min faster. This confirms the feasibility and effectiveness of applying the algorithm to the temperature control of the Earth simulator.

### 3.3. Temperature Control Module Design

#### 3.3.1. Electric Heat Film Heating Module

The main body of the Earth hot plate is designed with an aluminum disc because of its low density and because it is easy to use on the turntable and has good heat transfer performance; it is also easy to ensure uniform temperature when heating. The front surface of the hot plate is treated with a black anodic oxide coating to improve the emissivity, and the rear surface is an electric heating film. 

To ensure the temperature uniformity of the hot plate, the electric heating film is divided into two parts: the main heating film and auxiliary heating films. The main heating film is heated extensively to make its temperature reach the required temperature and to provide a constant temperature, while the auxiliary heating films ensure that the hot plate is heated evenly. In order to ensure the accuracy of the temperature control of the hot plate, multiple thermostats are adopted to achieve closed-loop control of the black body. That is, the heating film is divided into several uniform independent units, and the temperature of each unit is tested and set separately to make the hot plate warm up so that its temperature remains constant at a certain set value in the range of 35 °C~85 °C.

This method can manipulate the temperature of each part individually, which facilitates the completion of the hot plate temperature uniformity better than ±0.5 °C index. When the electric heating film stops heating, the hot plate is cooled down by the ambient temperature and the fans in front of the cold plate. And, due to the continuous detection of the temperature sensor, when the temperature does not meet the conditions, the hot plate is heated so as to achieve the purpose of controlling the temperature of the hot plate.

According to the theory of convective heat exchange in a small space, the heat exchange on the surface of the hot plate is determined by the following equation:

(7) $$q_{m}=\frac{k_{\varepsilon }}{b}(t_{h}-t_{c})$$

The Rayleigh coefficient $R_{a}$ associated with $q_{m}$ is:

(8) $$R_{a}=\frac{\beta g(t_{h}-t_{c})L^{3}}{va}$$

where a is the thermal conductivity of the hot plate, β is the expansion coefficient of the hot plate, L is the characteristic length, v is the material viscosity coefficient, $t_{h}$ is the temperature of the hot plate for radiation heat transfer, and $t_{c}$ is the radiation ambient temperature.

If the heat transfer coefficient is $k_{\varepsilon }$ and $k_{\varepsilon }$ = 0.035413 W/m·°C, the heat exchange Q is:

(9) $$Q=\frac{Ak_{\varepsilon }(t_{h}-t_{c})}{D}$$

where A is the area of the cold plate aperture (effective radiation area), $A=\frac{\pi D^{2}}{4}$, and D is the effective radiation diameter of the hot plate (determined by the cold plate).

The heating power required is:

(10) $$P=\frac{Ak_{\varepsilon }(t_{h}-t_{c})m_{hot}dt}{Dd\tau }$$

where $m_{hot}$ is the mass of the hot plate, dt is the adjustable range of the hot plate temperature, and dτ is the heating time.

According to the heating requirements, the heating film heating power can be calculated to be about 70 W, and the appropriate heating film can be selected.

#### 3.3.2. Semiconductor Cooling Module

A TEC semiconductor cooling chip is a device based on the thermoelectric effect, which has the advantages of easy control, low thermal inertia, a fast cooling rate, and high power [19]. Its basic element is a thermocouple; the formation of multiple P–N junctions in series forms a thermocouple. Furthermore, an insulating ceramic sheet is connected to both ends of the thermocouple to form the most basic semiconductor cooling sheet. In the semiconductor cold junction, the current flows from the N to the P, where the temperature drops and heat is absorbed from the environment; in the hot junction, the current flows from the P to the N, and the temperature rises, releasing heat. 

**Figure 9:**
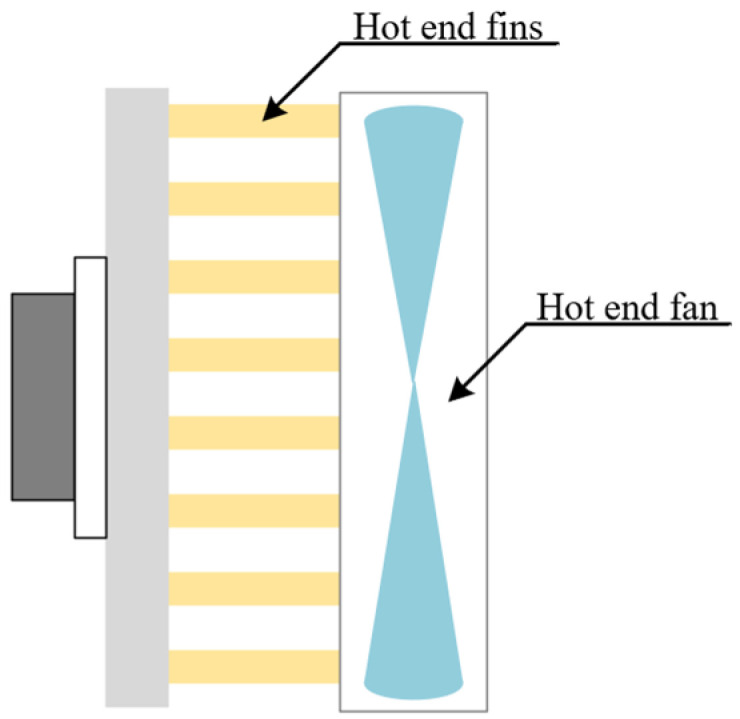
Hot-end air cooling diagram.

The cold end of the cooling chip is attached to the Earth cold plate to cool it down. The installation of cooling fins and fans for forced cooling at the hot end can ensure heat dissipation at the hot end with the aim of improving the efficiency of the cooling chip, as shown in Figure 9 [20]. The hot-end fins are made of aluminum, which has good heat transfer and is made into a dense tooth design to increase the contact area with the air. Then, by setting a fan to speed up the air flow, this helps a lot with heat dissipation. 

**Figure 10:**
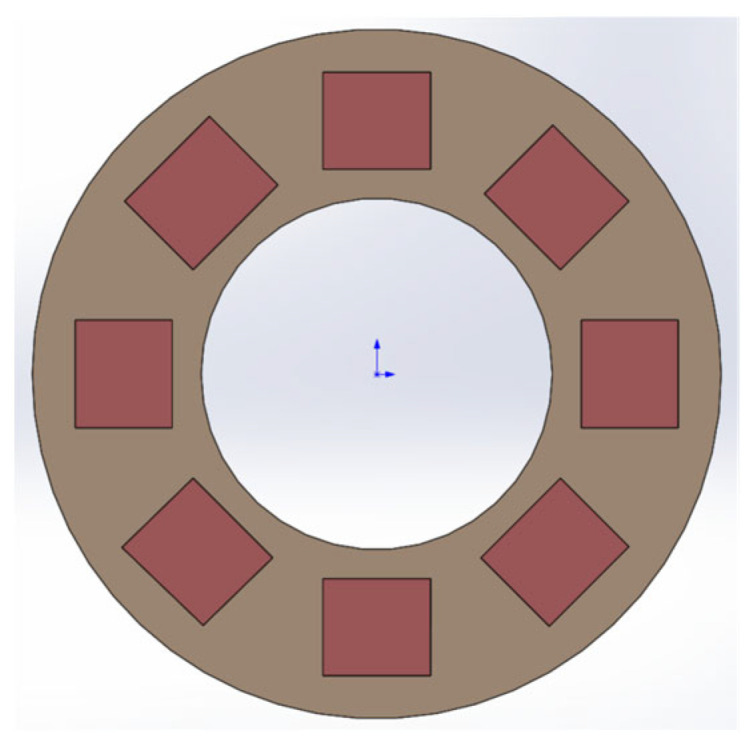
Semiconductor chip position.

The temperature control system of the Earth simulator controls the temperature of both the hot and cold plates so that the temperature difference between the two is similar to the difference in radiance between the Earth and space during the operation of the satellite. The back of the cold plate has a heat source provided by the hot plate, so there is no need to consider heating, only cooling. Furthermore, adding heat insulation film to the back of the Earth cold plate reduces the impact of the hot plate temperature. For cooling, we use the TEC semiconductor cooling ring method, as shown in Figure 10. By constantly checking the temperature of the cold plate, the power of each cooling plate is controlled to obtain the desired temperature. And, it is well integrated with the Earth simulator by using the embedded precise control method. 

Under general operating conditions, let the temperature of the cold end of the cooling chip be $T_{c}$, and the magnitude of its cooling coefficient does not change due to the change in temperature [21]. If the heat generated by the Thomson effect is neglected, then according to the first law of thermodynamics, we can obtain:

(11) $$Q_{h}=Q_{c}+P$$

The Peltier heat generated at the cold end of the semiconductor, i.e., the ideal cooling capacity, can be expressed as:

(12) $$Q_{p,c}=\pi I=(\alpha_{P}-\alpha_{N}IT_{C})=\alpha_{PN}IT_{C}$$

Due to the existence of irreversible Joule heat in the circuit, some heat will flow to the cold end of the cooling chip in the closed circuit, so the heat generated by Joule heat conduction should be removed from the ideal cooling capacity, i.e., the actual cooling capacity is:

(13) $$Q_{c}=Q_{p,c}-\frac{1}{2}Q_{J}=\alpha_{PN}IT_{C}-\frac{1}{2}I^{2}R$$

where $Q_{J}$ is the irreversible Joule heat.

Then, the actual heat dissipation of the cooling chip is:

(14) $$Q_{h}=Q_{p,h}+\frac{1}{2}Q_{J}=\alpha_{PN}IT_{C}+\frac{1}{2}I^{2}R$$

Taking into account the cooling capacity and the required drive requirements, the maximum temperature difference between the hot and cold ends, the installation size, and other factors, we selected the TEC1-19908 model, and its specific parameters are shown in Table 1.

## 4. Temperature Control System Simulation Results

**Figure 11:**
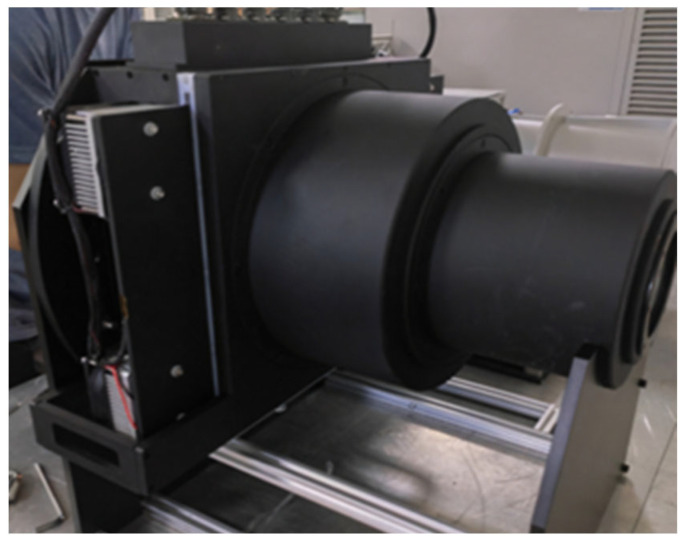
Actual Earth simulator.

To implement the simulator temperature control system, this paper uses ANSYS Workbench software for temperature analysis of the Earth cold plate and shows the effect of the Earth hot plate temperature on the temperature of the cold plate through the temperature map. For better temperature analysis, we built a simulation model based on the actual Earth simulator, as shown in Figure 11. 

**Figure 12:**
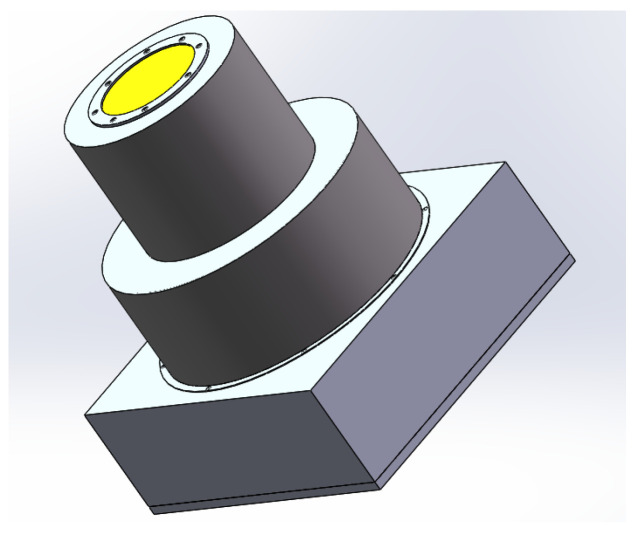
Earth simulator model.

During the modeling process using SOLIDWORKS (2017), the model was appropriately simplified in order to reduce computer workload and to improve computing efficiency, and the final result is shown in Figure 12. 

**Figure 13:**
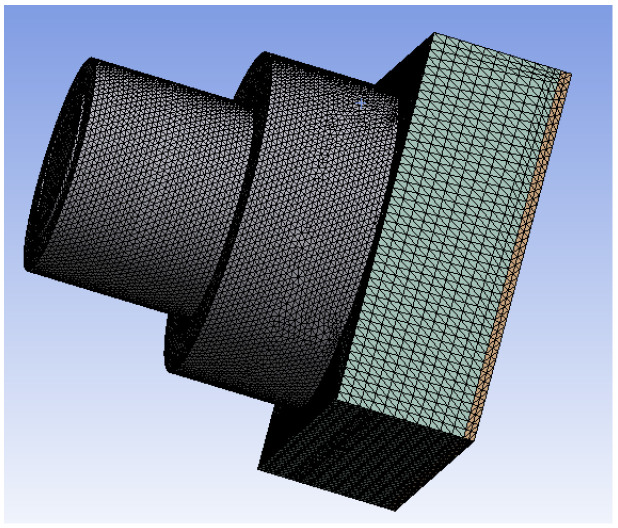
Earth simulator meshing.

Import the model made with SOLIDWORKS into ANSYS Workbench and define the material properties of the model. The model as a whole is made of aluminum alloy, and the surface of the metal disc is black anodized to improve the surface emissivity and to make it a hot plate with uniform heat dissipation. The meshing of the model is shown in Figure 13. The mesh division has a decisive influence on the final structural analysis, so it is important to ensure the quality of the divided mesh cells. Therefore, we ensure that the quality of the divided grid cells is >0.3.

**Figure 14:**
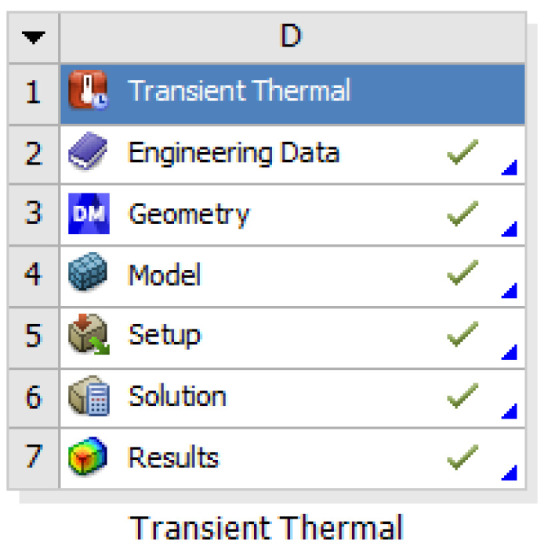
ANSYS transient heat transfer model.

The temperature field using the transient heat transfer model is shown in Figure 14; set the ambient temperature (initial temperature) to 20 °C for the back of the hot plate (heating film location) to add the temperature required for the experiment (85 °C added in the experiment). And, add heat flow to the backside of the cold plate by the above calculation to mimic the gas heat transfer between the hot plate and the cold plate. The time of the experiment was stretched to observe the effect of heating the hot plate from the ambient temperature to the maximum temperature and the temperature of the hot plate stabilized at 85 °C on the cold plate, respectively. 

First, when the semiconductor cooling chip is not working, determine the refrigeration chip working environment temperature (only air cooling). Convection was added to the location of the cooling chip in ANSYS Workbench to simulate the forced air cooling device described above. The convection coefficient is about 100 W/m^2^·°C. 

**Figure 15:**
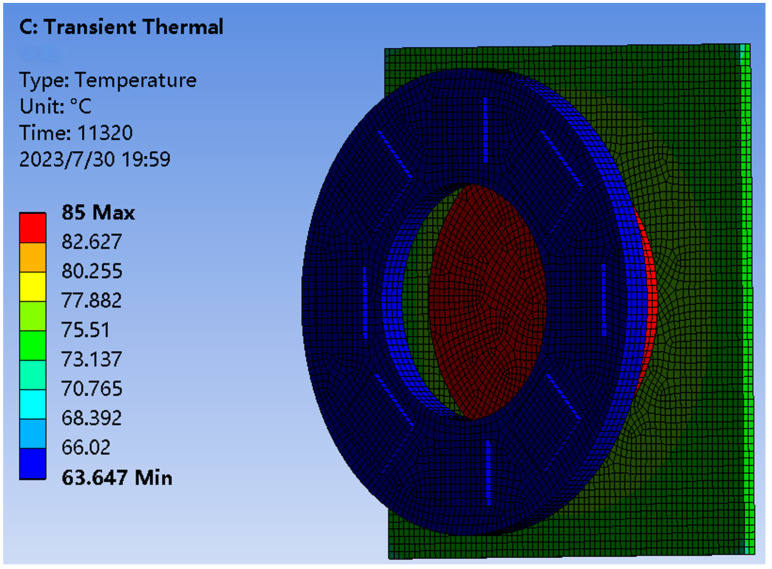
Air cooling temperature impact.

After the heating process has gradually stabilized and the heating and cooling links in the Earth simulator have stabilized (about 5 min), we can view the temperature graph, as shown in Figure 15. The temperature of the cold plate is maintained roughly at 62.632 °C. According to the universal working temperature of the semiconductor cooling chip, which is 70 °C maximum, it is known that the cooling chip can work normally in the environment of the Earth simulator. 

**Figure 16:**
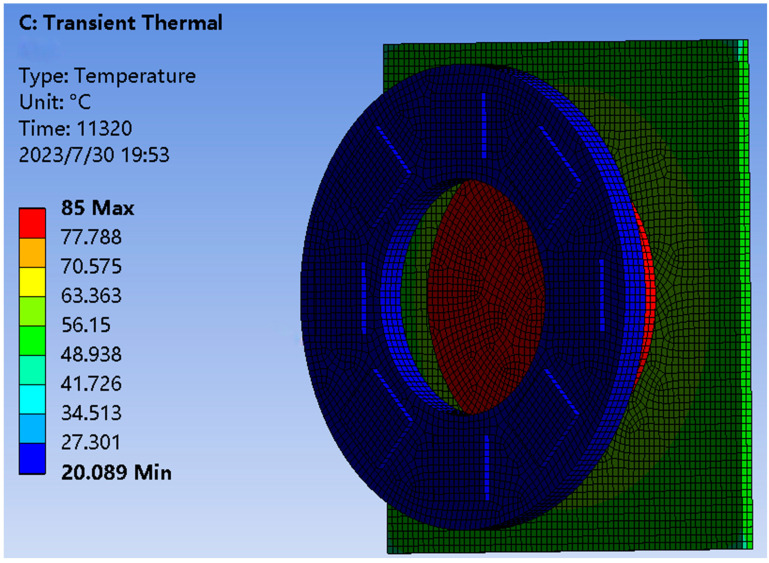
Overall temperature impact.

Finally, the cooling capacity of the semiconductor cooling chip is calculated and added to the cooling chip. The air cooling device and the cooling chip were made to work simultaneously, and the results obtained are shown in Figure 16. The results show that the semiconductor cooling inside the Earth simulator controls the temperature of the cold plate at about 20.089 °C to meet the temperature control requirements. 

**Figure 17:**
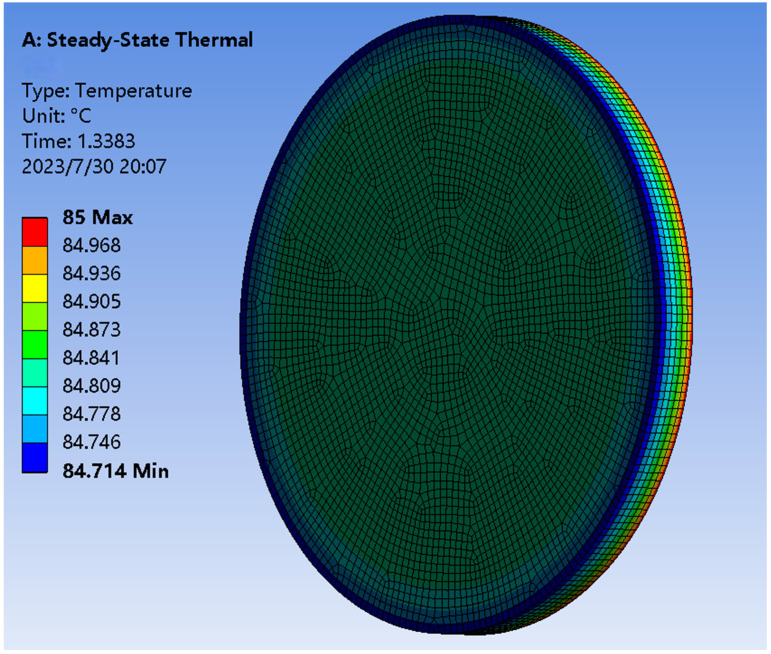
Earth hot plate temperature uniformity.

In order to make the image received by the Earth sensor clear and to reduce the measurement error [22], the Earth needs to be observed as a uniformly heated disk so that the temperature uniformity at the edge of the disk is better than ±0.5 °C. The temperature analysis shown in Figure 17 shows that the overall temperature uniformity of the hot plate is better than ±0.3 °C when the heating film is heated on the back of the hot plate to keep its temperature at 85 °C. It is higher than the index requirement and satisfies the test conditions of the Earth sensors.

## 5. Conclusions

In this paper, a temperature control system based on semiconductor cooling technology combined with air cooling is designed for the infrared Earth simulators for a turntable. The temperature range of the Earth hot plate to be controlled is calculated by the Earth radiation; a fuzzy PID-based control algorithm is adopted to improve the accuracy of the Earth simulator. The feasibility of the design results for the temperature control system is confirmed by software simulation, and the technical index requirements can be met. Finally, the overall structure of the Earth simulator is designed to achieve miniaturization and portability of the Earth simulator with guaranteed accuracy. New technologies are provided for the development of Earth simulators.

This system also has some limitations, such as the higher noise of the air cooling, higher power consumption, and the need for regular testing or replacement of the cooling chip sheet. In the future, we will explore new cooling methods to minimize noise and combine advanced sensing technologies and feedback algorithms to conserve resources. In addition, we will conduct more extensive testing and optimization of semiconductor cooling combined with air-cooling technology for a wide range of applications in different types and sizes of infrared Earth simulators. The Earth simulator will be placed on a five-axis turntable to calibrate and test specific data on the increased accuracy of the Earth sensors. As aerospace technology continues to evolve, satellites will place increasing demands on the Earth sensors they carry.

## Figures and Tables

**Table 1:** TEC cooling film type and each parameter.

TEC Model	Max. Temperature Difference Current (A)	Max. Temperature Difference Voltage (V)	Max. Temperature Difference (°C)	Max. Cooling Power (W)	Dimension (mm)	Temperature Use Range (°C)
TEC1-19908	8.0	24.6	68	111	40 × 40	−60~200

## Data Availability

Not applicable.
